# Volterra Kernel Estimation of White Light LEDs in the Time Domain

**DOI:** 10.3390/s18041024

**Published:** 2018-03-29

**Authors:** Grzegorz Stepniak, Marcin Kowalczyk, Jerzy Siuzdak

**Affiliations:** Institute of Telecommunications, Warsaw University of Technology, Nowowiejska 15/19, 00-665 Warsaw, Poland; m.kowalczyk@tele.pw.edu.pl (M.K.); siuzdak@tele.pw.edu.pl (J.S.)

**Keywords:** visible light communications, nonlinearity, advanced modulation formats

## Abstract

In this paper, we present a time domain method for extracting coefficients of nonlinear Volterra-series kernels for white light-emitting diodes (LED) used both for illumination and visible light communications. We show that this method may have several advantages over the thus far more popular frequency domain method. We successfully apply the measured kernel coefficients up to the 3rd order for the modeling of nonlinear distortion impact on advanced modulation formats: pulse amplitude modulation, carrierless amplitude phase and orthogonal frequency division multiplexing. The impact of blue filtering on dynamic nonlinearity is also presented.

## 1. Introduction

Light-Emitting Diode (LED) communication is an attractive and low-cost solution for free space communication systems [1] and transmission in polymer optical fibers (POF) [2]. Recently, due to the growing market share of LEDs in lighting applications, they have attracted increased attention in the context of visible light communications (VLC). The idea of VLC is to use lighting LEDs both for illumination and for distribution of high-speed data signal. VLC is now considered a technology complementary to 5G mobile systems, as it can provide additional high-capacity communications in the so-called optical attocells, where the receiver is directly illuminated by the white light source [3]. There are two types of LEDs used for lighting applications. The first is a blue chip covered with a phosphorous layer, serving as a blue to yellow color converter. Both colors combined form light perceived as white. Unfortunately, the phosphorous layer has a slower time response and typically limits the bandwidth of LED to a few MHz. This can be managed with receiver blue filtering [4] or digital equalization. Unfortunately, both solutions impose either power or noise enhancement penalties [5,6]. This problem is avoided in the second kind of lighting LED, which consists of three or more chips of different colors (e.g., RGB). In addition, as different chips can be modulated independently, wavelength division multiplexing can be used, which increases the data rate by a factor of channel number. 

Regardless the LED type, LEDs are nonlinear devices, i.e., the emitted optical signal power a nonlinear function of the modulating current. This nonlinearity can have both static and dynamic characteristics. The former is mainly caused by efficiency drop, i.e., decreased internal quantum efficacy with increasing injection currents [7]. The latter is caused by difference in carrier lifetimes, depending on the modulating current [8]. Unfortunately, unlike inter-symbol interference (ISI), nonlinearity cannot be overcome by well-established methods of linear equalization and may become the major transmission rate limiting factor, especially for spectrally efficient advanced modulation formats, which require a high signal-to-noise ratio (SNR) at the receiver. Accurate description of LED nonlinearity is a vital issue, as it is necessary to estimate the information capacity of the link, and it can help evaluate the performance of different modulations. While the nature of nonlinearity has already been described in carrier rate equations [8], this simple description does not accurately model the dynamics of the device [9]. Therefore, description of LED nonlinearity as a black-box system could have a greater significance for LED transmission system modeling than theoretical models based on carrier transport [9]. In our approach, LED input/output relation is represented with Volterra series, which is a general description of nonlinear systems behavior. The Volterra model has already been used for LED nonlinearity identification [9,10,11] and has also successfully been applied to LED nonlinearity compensation for single-carrier [12] and multicarrier modulation formats [13].

## 2. Theory

To properly describe LEDs with the Volterra series, the series coefficients need to be measured first. Here, two methods can be applied: the frequency domain and the time domain methods. In the frequency domain method, the device is probed with a current waveform $i\left(t\right)=I_{1}\cos\left(2\pi f_{1}t\right)+\ldots +I_{N}\cos\left(2\pi f_{n}t\right)$ and the amplitudes of harmonics generated at frequencies of $f_{1}\pm f_{2}\pm \ldots \pm f_{n}$ are measured, which can be related to the *n*-th order Volterra kernel $\left|H_{n}\left(\pm f_{1},\;\ldots ,\;\pm f_{n}\right)\right|$. In this method, *n* tones are needed to estimate the kernel up to the *n*-th order. This approach has been applied to estimate the Volterra kernel of LEDs up to the 2nd [9,10] and 3rd [11] order. The frequency domain method has some limitations, though. Firstly, only the magnitude response of the kernel can be measured. Secondly, it cannot measure the response at the kernel diagonals, e.g., $H_{2}\left(f_{1},-f_{1}\right)$, as the intermodulation product of this component falls at dc. Thirdly, for some frequencies of the probing harmonics, intermodulation products of the 2nd and higher orders fall at the same frequencies. For example, if two tones at frequencies *f*_1_, 2*f*_1_ are used, the 2nd order intermodulation products fall at *f*_1_ and 3*f*_1_, and the third order at 3*f*_1_, 4*f*_1_, 6*f*_1_. It is readily visible that the tone at *f*_1_ is disturbed by the presence of the probing signal, while the tone at 3*f*_1_ is interfered with by the presence of the 3rd order distortion. To avoid this, the kernels need to be separated by linear regression. In this method, the probing is done with tones at various amplitudes and the dependence of the amplitudes of the intermodulation products of different orders on the amplitude of probing signal is exploited as an additional degree of freedom in separating the kernels [12]. Unfortunately, this procedure highly complicates the measurement. 

The time domain method is based on the relation between the input training signal *x*(*t*) and the output signal *y*(*t*) as described with time Volterra series [12]

(1) $$y\left(t\right)=\overset{M}{\underset{m=0}{\sum }}h_{m}\left(\tau_{1},\ldots ,\tau_{m}\right)⨂x\left(t\right)+n\left(t\right),$$

where $h_{m}\left(\tau_{1},\ldots ,\tau_{m}\right)$ is the Volterra kernel of *m*-th order in the time domain at *τ_m_* time delay, *n*(*t*) is additive noise signal, *M* is the number of kernel orders and

(2) $$h_{m}\left(\tau_{1},\ldots ,\tau_{m}\right)⨂x\left(t\right)=\int \ldots \int h_{m}\left(\tau_{1},\ldots ,\tau_{m}\right)x\left(t-\tau_{1}\right)\ldots x\left(t-\tau_{m}\right)dx_{1}\ldots dx_{m}.$$

We assume the probing signal to be a *M*-ary filtered pulse amplitude modulation (PAM) waveform at baud rate *r* and symbol interval *T_s_* = 1/*r*. The time delays in (2) are then quantized at discrete multiples of *T_s_* and the equation can be transformed into a discrete form

(3) $$y\left(n\right)=h_{0}+\overset{M}{\underset{m=1}{\sum }}\overset{M_{1}-1}{\underset{i_{1}=0}{\sum }}\ldots \;\overset{M_{m}-1}{\underset{i_{m}=0}{\sum }}h_{m}\left(i_{1},\ldots ,i_{m}\right)x\left(n-i_{1}\right)\ldots x\left(n-i_{m}\right)+n\left(t\right),$$

where *M_m_* is the memory length of the *m*-th order. As a prerequisite of the estimation, the maximum kernel order has to be assumed. It is a tradeoff between the estimation accuracy and complexity, as the number of series terms is given roughly as the memory length of the kernel to the power of the kernel order. However, the symmetry of the kernel coefficients can be exploited to reduce the number of terms that need to be estimated. For example, from (3), it is readily visible that $h_{2}\left(i_{1},i_{2}\right)=h_{2}\left(i_{2},i_{1}\right)$. The total number of irredundant terms is

- $M_{1}$
- $\frac{M_{2}\left(M_{2}+1\right)}{2}$
- $\left(\begin{array}{c} M_{3}+3\\ 3 \end{array}\right)-\left(\begin{array}{c} M_{3}+2\\ 2 \end{array}\right)$

in the 1st, 2nd, and 3rd order, respectively [14]. After some rearrangements, (3) can be expressed in a matrix formalism

(4) Y(n)=X(n)H+N(n)

where ***Y***(*n*) = [*y*(*n*),*y*(*n* − 1)...,*y*(*n* − *L*)]*^T^*, ***N***(*n*) = [*n*(*n*),*n*(*n* − 1)...,*n*(*n* − *L*)]*^T^* and

(5) $$H={\left[h_{1}\left(0\right),h_{1}\left(1\right),\ldots ,h_{1}\left(M_{1}\right),h_{2}\left(0,0\right),h_{2}\left(0,1\right),\ldots ,h_{2}\left(M_{2},M_{2}\right),\ldots \right]}^{T}$$

is a vector containing the Volterra coefficients, and $X={\left[X_{1},X_{2},\ldots ,X_{M_{1}},X_{M_{1+1}},X_{M_{1}+M_{2}+\ldots }\right]}^{T}$ is the training sequence matrix, with the following combinations of the training symbols *x*(*n*):

(6) $$X_{1}={\left[x\left(n\right),x\left(n-1\right),\ldots ,x\left(n-L\right)\right]}^{T}\;X_{2}={\left[x\left(n-1\right),x\left(n-2\right),\ldots ,x\left(n-L-1\right)\right]}^{T}$$

$$X_{M_{1}+1}={\left[x\left(n\right)x\left(n\right),\;x\left(n\right)x\left(n-1\right),\ldots ,x\left(n\right)x\left(n-L\right)\right]}^{T},$$

where *L* is the length of the training sequence. The coefficients ***H*** can be sought using least squares (LS) solution [15]

(7) $$H={\left(X^{T}X\right)}^{-1}X^{T}Y.$$

It is noted that by doubling the training sequence length the variance of estimated coefficients is reduced by half. As an alternative to the LS method, recursive least squares (RLS) or least mean squares (LMS) adaptive algorithms may be applied to find ***H*** [16], which could help to avoid desynchronization if the transmitter and receiver are using different clocks. 

**Figure 1:**
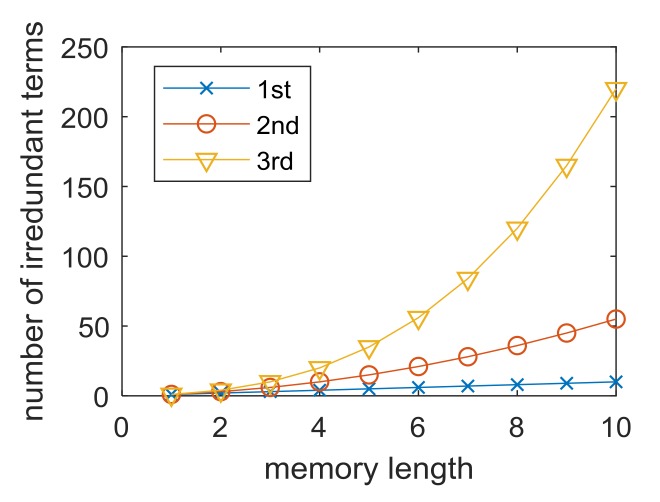
The number of irredundant terms of each Volterra order.

We can now briefly comment on the numerical complexity of the estimation. The number of irredundant terms for each order has been plotted in Figure 1 for varying values of the memory parameter. Clearly, the numerical complexity grows rapidly with the inclusion of higher-order nonlinearity. In addition, computation of each of the elements of ***X*** matrix requires (*O*-1) multiplications, where *O* is the order number. Finally, the LS problem (7) is most efficiently solved by means of QR decomposition of matrix ***X***, which requires approximately $2L\times {\left(M_{1}+M_{2}+M_{3}\right)}^{2}$ flops [17]. 

The time domain coefficients can be transformed into the frequency domain using the Fourier transform [14]

(8) $$H\left(f_{1},\ldots ,f_{n}\right)=\int \int h_{n}\left(\tau_{1},\ldots ,\tau_{n}\right)e^{\;-\;j2\pi \left(f_{1}\tau_{1}+\ldots +f_{n}\tau_{n}\right)}d\tau_{1}\ldots d\tau_{n}.$$

The time domain method has certain advantages over the frequency domain. As it yields complex valued coefficients, it allows for the prediction of the signal at the output of the model for known input signals. It does not require estimation at different modulating current values to separate the overlapping products of different kernels. Finally, the measurement procedure is instant in a setup with a PAM signal generator and digital storage oscilloscope (DSO). In the same manner as in the frequency domain method, the nonlinear distortions coming from higher-order terms, not included in the estimation, bias the estimated coefficients. 

## 3. Experimental Setup and Measurement Procedure

**Figure 2:**
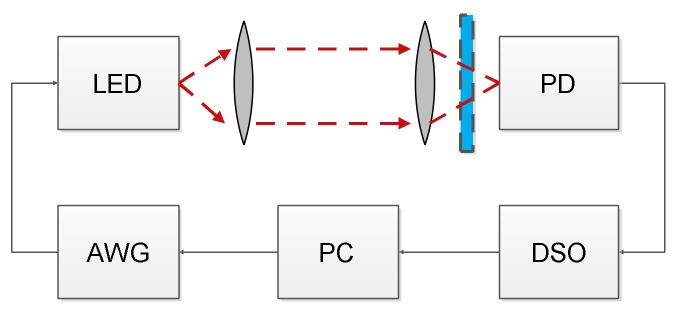
Measurement setup. AWG—arbitrary waveform generator, PD—photodetector, DSO—digital storage oscilloscope, PC—personal computer.

**Figure 3:**
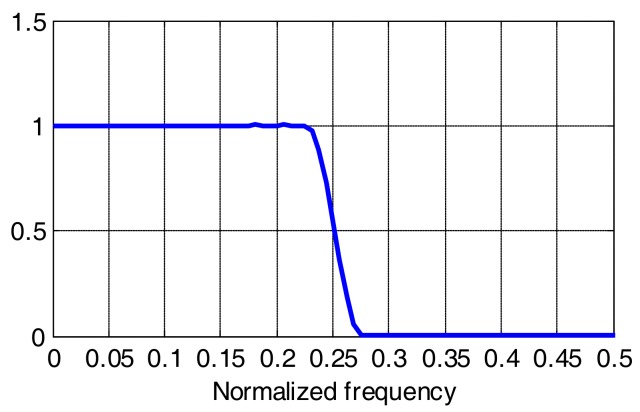
RRC filter response for roll-off 0.1.

The experimental setup for the time domain method requires a multilevel random signal generator and digital storage oscilloscope (DSO) for recording the output signal. The setup schematic is shown in Figure 2. First, random symbols of the PAM-8 signal are generated offline in Matlab and upsampled with a factor of 2. Next, we apply a root raised cosine (RRC) filter with 0.1 roll-off coefficient to shape the signal spectrum to quasi-rectangular. The choice of the roll-off is a tradeoff between spectrum flatness, total symbol duration and peak to average power ratio (PAPR), which increases for lower roll-off values. At this value, the filter spectrum is flat up to approx. 0.9*r*/2 (Figure 3). The generated PAM signal is fed into AWG, which modulates the LED. The modulation index is different for different LEDs and scenarios. After short transmission in free space, the signal is photodetected and sampled in DSO. In the case of white phosphorescent LEDs, blue filtering is applied at the receiver. Further processing in Matlab involves resampling to 2*r*, synchronization using the cross-correlation method, filtering with a matched RRC filter, averaging over all copies of the received sequence captured in one DSO frame (approx. 30) for additive Gaussian noise cancellation, downsampling to symbol frequency and LS estimation, as described above. It is noted that in the applied method, the received signal was AC coupled, so the estimated kernel depends on the bias current of the LED. 

The applied time domain method also has some restrictions related to the spectral extent of the estimated kernel. As governed by the sampling theorem, the linear kernel (frequency response) can be measured up to *r*/2 (in addition, a 10% margin on the RRC response should be assumed). However, further restrictions to higher-order terms will apply. We illustrate this for the 2nd order kernel. Let us consider a LED probed with a single tone at frequency *f*_1_ that will generate a 2nd order harmonic at 2*f*_1_. This harmonic falls outside the RRC filter bandwidth at the receiver, and hence *H*(*f*_1_*,f*_1_) cannot be measured. It is noted that RRC filtering is necessary, as this harmonic would otherwise cause aliasing at frequency *r* − 2*f*_1_. Therefore, the 2nd order can be fully measured only up to |*f*_1_ *+ f*_2_|< *r*/2 frequency. In general, the *M*-th order kernel can be estimated up to *r*/(2*M*) frequency. 

It is noted that we assume that the LED is the dominating source of both the bandwidth limitation and nonlinearity in the system. The bandwidth of the detector was almost order of magnitude higher. We made sure that the detector is placed at the optimum distance from the transmitter to make sure that it is far from being saturated.

## 4. Measurement Results and Verification

### 4.1. Phosphorescent White Light LED 

**Figure 4:**
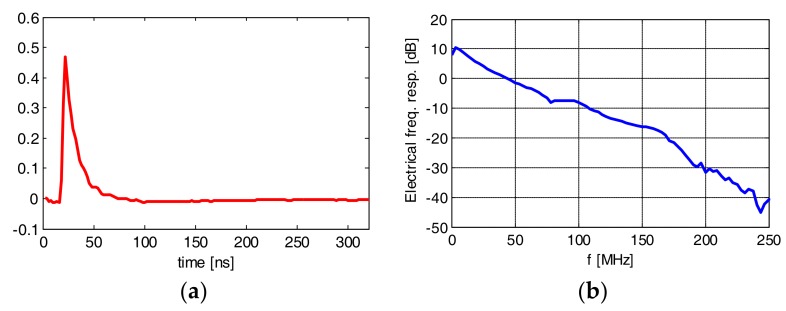
Impulse (**a**) and frequency (**b**) responses of LE UQ Q9WP transmission setup with blue filtering.

**Figure 5:**
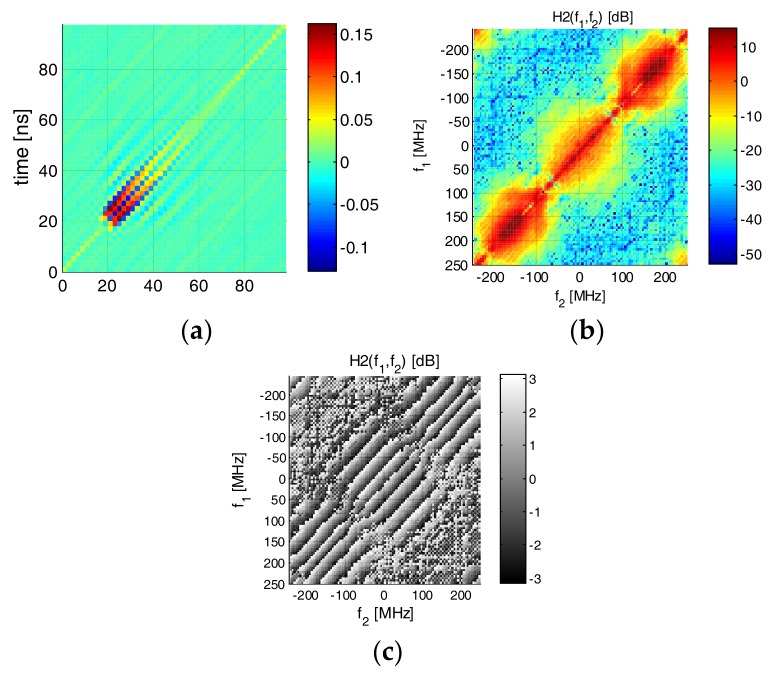
Second order kernel of LE UW Q9FP: (**a**) time domain; (**b**) frequency domain magnitude in dB scale; (**c**) phase in radians.

**Figure 6:**
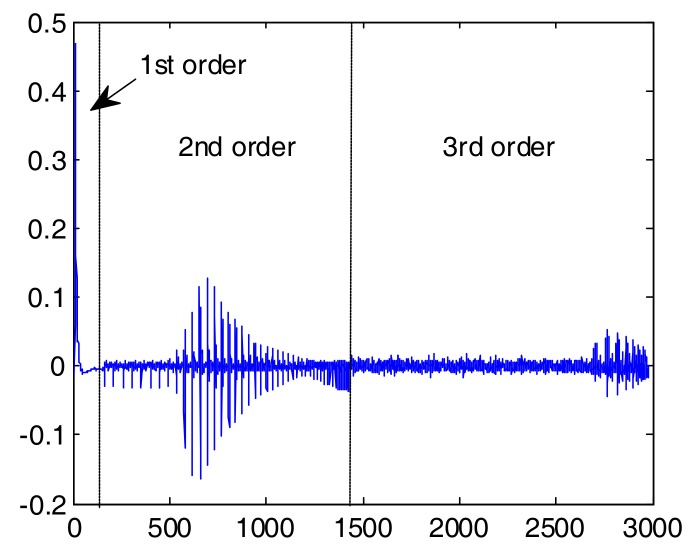
Vector ***H*** as defined in (Equations (5)–(7)). Relation of amplitudes in different nonlinearity orders. Coefficient index on the x-axis.

Here, we tested an Osram LE UQ Q9WP LED. It is noted that the LED was equipped with a driving circuit with a current amplifier. The probing signal consisted of 20 k symbols of PAM-8 modulation transmitted at 500 Mbaud. In the case of this LED, the modulation index was close to 100%. We assumed estimation with memory length (in symbols) of 160, 50 and 20 for the 1st, 2nd and 3rd nonlinearity orders, respectively. The impulse and frequency response (linear kernel) is shown in Figure 4. The 6 dB (electrical) bandwidth of the device is in the order of 20 MHz. The 2nd order time kernel is shown in Figure 5a. It can be seen that most of the non-zero coefficients in the time domain are concentrated along the diagonal, and the maximum significant delay difference between τ_1_ and τ_2_ is in the order of 10 ns. The frequency domain 2nd order kernel is shown in the same Figure 5b. Most of the 2nd order distortion is present at the lowest frequencies; however, a significant amount of distortion is visible in the (−100,100) MHz region. We attribute this distortion to the LED-driving circuit. For completeness, we have also shown the phase response of the 2nd order kernel (Figure 5c). In Figure 6, the ***H*** vector as defined by (Equations (5)–(7)) has been plotted to demonstrate the relation in magnitude between different kernels. The amplitudes of coefficients in higher-order kernels decrease with the kernel order (but obviously their number raises). 

### 4.2. Applying the Measured Kernel to Predict LED Behavior for Advanced Modulation Formats

The measured Volterra kernels are universal in the sense that once measured for PAM-8, they should predict the nonlinear distortion effect of this LED on different modulation formats under the same experimental conditions. To verify this, we transmitted signals of different advanced modulations: PAM-4 signal, carrierless amplitude-phase (CAP)-16 and orthogonal frequency division multiplexing (OFDM) signal with quadrature amplitude modulation (QAM)-4 at all subcarriers. Next, we averaged the received signals over the received copies to reduce the additive white noise and compared with signals synthesized using the Volterra kernel estimated in Section 4.1. As a similarity indicator, we evaluated the variance of the error between reconstructed $\hat{y}\left(n\right)$ and received y(n) signals

(9) $$\epsilon =E\left\{\left(y\left(n\right)-\hat{y}(n\right){)}^{2}\right\}.$$

To avoid resampling errors, as the estimation was performed for 500 Mbaud, the sampling frequencies (or baud rates) of the predicted signals were 500, 250 or 125 Mbaud. The results are presented in Table 1. The highest reduction of the approximation error was obtained for the 500 Mbaud PAM-8 signal after including the 3rd order kernel (7 dB). It is not surprising as this signal was used for the measurement, and only in this case was there a perfect synchronization between the transmitter and receiver. For the remaining signals, the estimated kernel in the time domain can be time shifted up to half a symbol period with respect to the measured signal, which imparts the reconstruction. In all cases, error reduction was observed, the highest after including the 2nd order kernel. In one case (PAM-4, 500 Mbaud), the 3rd order kernel slightly increased the error.

**Figure 7:**
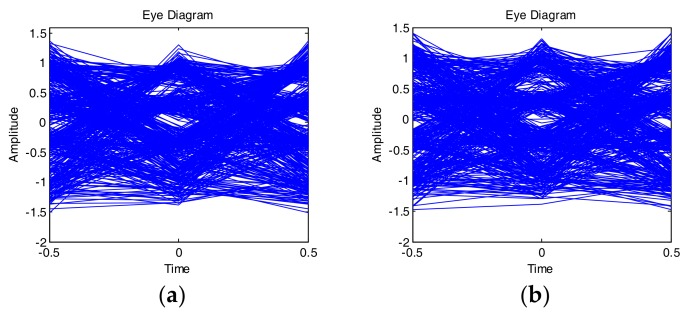
Eye diagrams after DFE for 250 Mbaud PAM-4 signal. Received (**a**) and synthesized (**b**).

**Figure 8:**
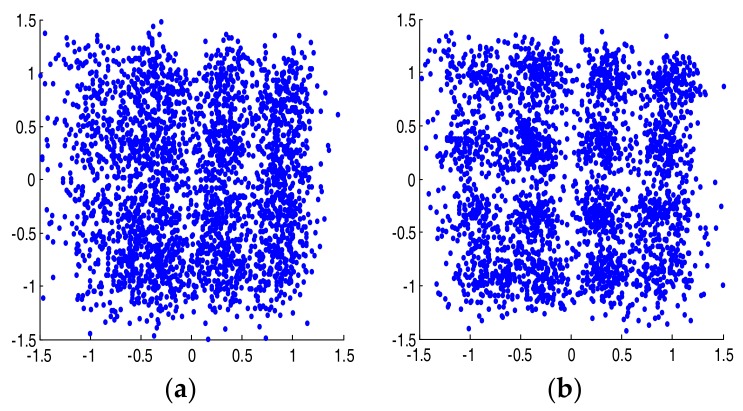
Received constellations after DFE for 125 Mbaud CAP-16 signal. Received (**a**) and synthesized (**b**)

In the next step, we compared eye diagrams of the received and reconstructed signals obtained after decision feedback equalizers (DFE) with 30 forward and 10 backward taps. The results for PAM-4 and CAP-16 are presented in Figure 7 and Figure 8, respectively. The similarity between the eye diagrams and constellations is readily visible. It is noted that without including the 2nd and 3rd order terms in the reconstruction, the DFE was able to compensate the ISI completely and the eye diagrams and constellations of the synthesized signals were perfectly undistorted. It cannot deal with nonlinear distortion, though, which is the main source of quality degradation in Figure 7 and Figure 8.

**Figure 9:**
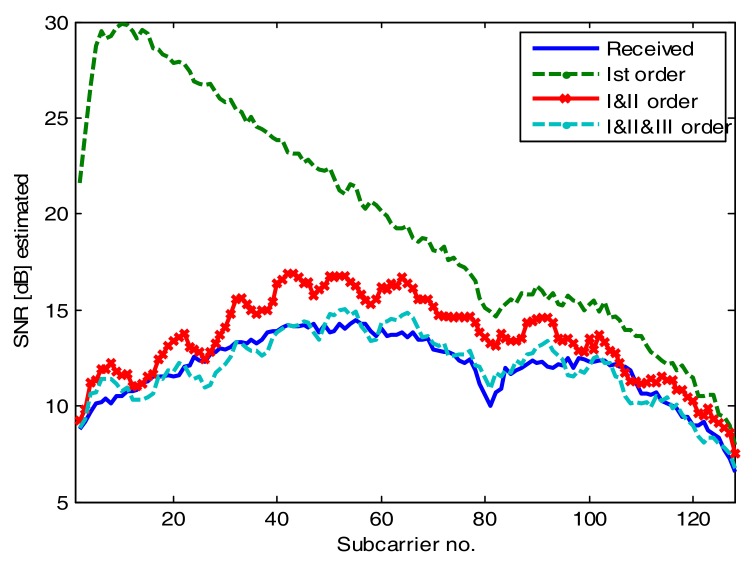
Signal to interference and noise ratio (SINR) for OFDM-QAM-4 transmission for all subcarriers.

Particularly interesting is the case of the OFDM signal, where the spectral distribution of the distortion can be extracted. We transmitted an OFDM signal loaded with QAM-4 modulation at all subcarriers. Upon reception and single-tap equalization, we estimated the signal to interference and noise ratio (SINR) distribution among subcarriers. The transmitted signal had 500 symbols, the number of subcarriers was 128, and the cyclic prefix length was 30 samples to eliminate ISI. The frequency of the highest subcarrier was 125 MHz. As this time only 2 copies of the signal were captured in one oscilloscope frame, noise averaging was not performed, but additive noise of −22 dB with respect to the signal was added to the synthesized signal. In addition, we found that for OFDM, the power of the signal at the input to the Volterra kernel had to be increased by 8 dB with respect to the previous signals. This is to compensate for the PAPR of OFDM. The results are shown in Figure 9. As we can see, for the approximation with only the 1st kernel, the SINR follows the frequency response of the link. By adding the 2nd and 3rd kernel, we can almost perfectly model the effect of the nonlinearity on the OFDM signal over the whole spectrum of interest. This is despite quite a small error variance reduction indicated in Table 1. 

### 4.3. Impact of Optical Filtering on White LED Nonlinearity

**Figure 10:**
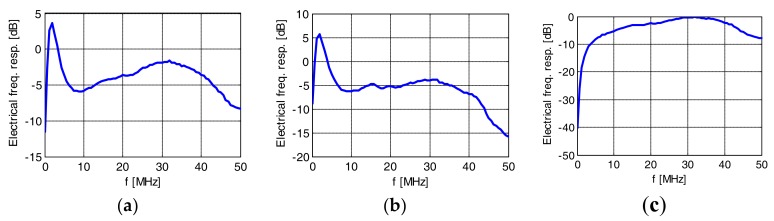
Frequency response of white (hot) LED without filter (**a**), with yellow (**b**), and blue filter (**c**) at the receiver.

**Figure 11:**
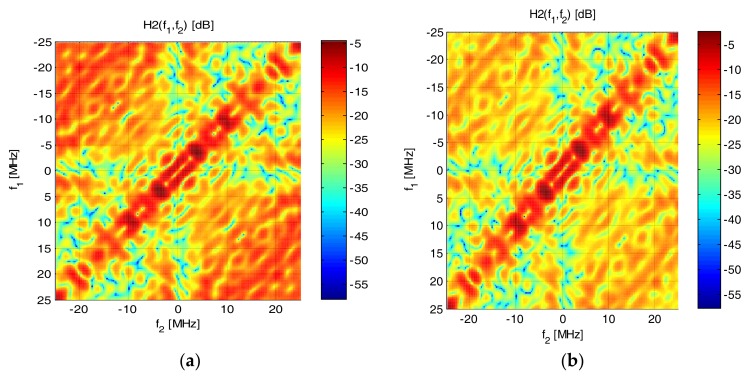

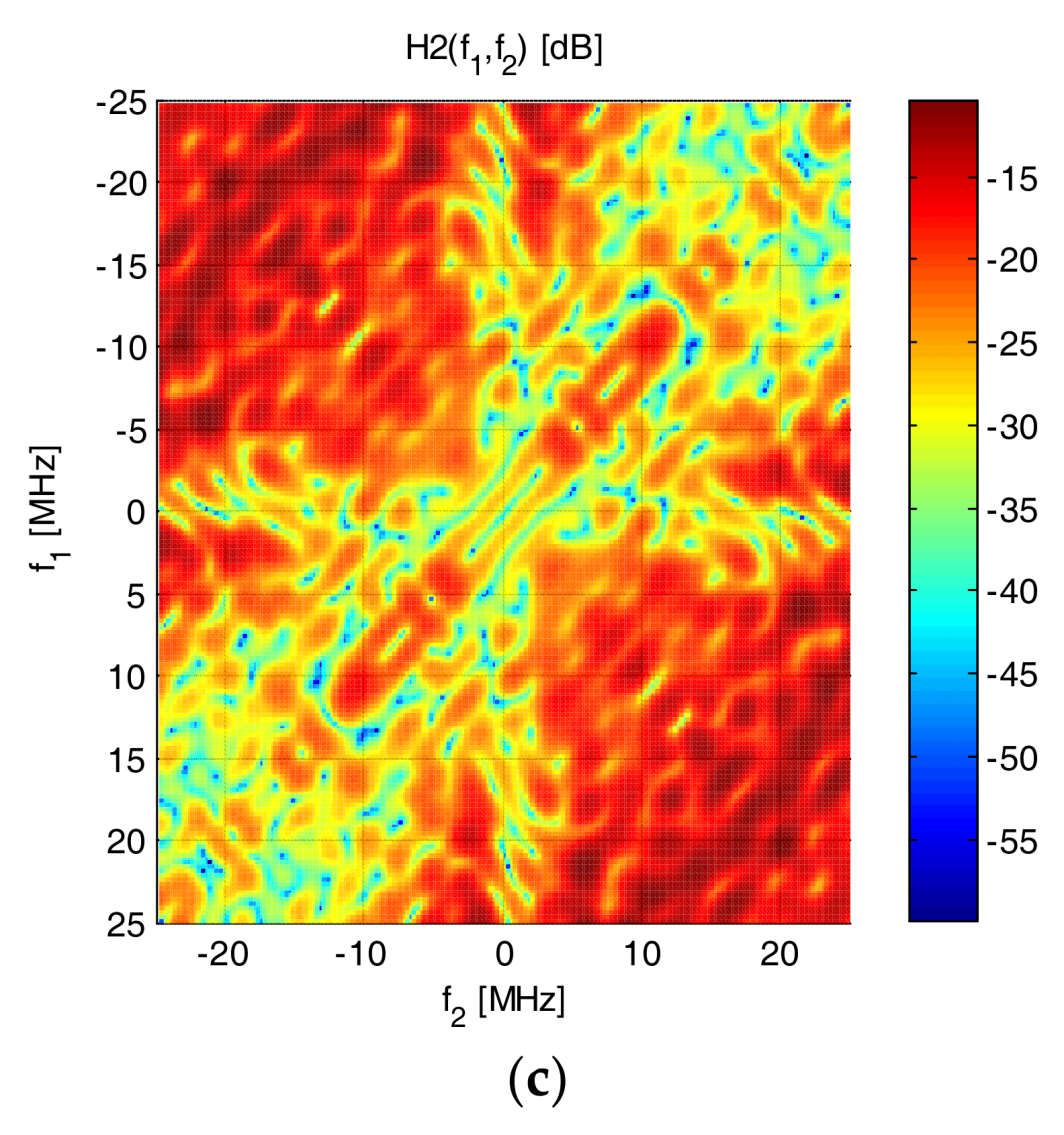
Frequency domain Volterra kernel of white (hot) LED without filter (**a**), with yellow (**b**), with blue filter (**c**) at the receiver.

In phosphorous white LED communications link receivers, a blue filter is typically applied to cut off the light generated in the slow-response phosphorescent layer and increase the electrical bandwidth of the system [3]. Although the impact of blue filtering on the frequency response is well known [5], to the best of our knowledge, it has never been studied in the context of nonlinear distortion magnitude and distribution. In this paper, we measured the Volterra kernel of a 1W Luxeon white phosphorescent LED of hot color temperature. The receiver was equipped with blue and yellow filters with their cut-off at 480 nm (this wavelength corresponds to the blue and yellow color boundary), and also without filter. The probing signal was transmitted at 100 Mbaud. The measured frequency responses are shown in Figure 10. The peak close to DC can be attributed to slow yellow light, and the plateau between 10 and 40 MHz to the blue light. Please note that to study the nonlinearity of this particular LED, we had to apply an additional electrical amplifier on the modulating current (Mini-Circuits 2FL-1000 H+), which cut out the spectrum from 0 to 2 MHz. The measured 2nd order Volterra kernels are shown (in the frequency domain) in Figure 11. The cross-like pattern close to DC of one of frequencies is attributed to the effect of the mentioned amplifier. It is readily visible that, in a similar manner as the 1st order frequency response, the 2nd order kernel also depends on optical filtering, with an increased impact of nonlinearity at low frequency when the blue filter at the receiver is not applied. Our study is not conclusive as to the origin of low-frequency nonlinearity, which may be a result of the fluorescence process, or may simply stem from the higher power at low frequencies when yellow or no filter is applied.

## 5. Conclusions

The Volterra series representation is a viable and practical method for nonlinear behavior description of white light LEDs in high-speed communications links. In this paper, we advocate for estimation of the LED’s Volterra kernel in the time instead of the frequency domain. We have shown that there are several advantages to this approach, including less complicated measurement procedure and full information on the kernel coefficients, which can be applied to modeling of advanced modulation formats performance in the VLC link. In particular, we have demonstrated that the Volterra kernel estimated using one modulation (in our case PAM-8) can be successfully used for nonlinear LED behavior modeling under other modulation types. 

## Figures and Tables

**Table 1:** Error variance between received and reconstructed signals for different number of kernels included (signal power normalized to unity).

Signal Type	1st	1st & 2nd	1st & 2nd & 3rd
PAM-8, 500 Mbaud ^1^	0.246	0.062	0.049
PAM-4 500 Mbaud	0.255	0.078	0.087
PAM-4 250 Mbaud	0.144	0.9	0.81
CAP-16 125 Mbaud	0.233	0.126	0.11
OFDM, N = 128, f_N_ = 125 MHz	0.11	0.089	0.08

^1^ same as used for estimation.

## References

[1] Vucic J., Kottke C., Nerreter S., Langer K.D., Walewski J.W. (2010). 513 Mbit/s Visible Light Communications Link Based on DMT Modulation of a White LED. J. Lightwave Technol..

[2] Zeolla D., Nespola A., Gaudino R. (2011). Comparison of different modulation formats for 1 Gbit/2 SI-POF transmission systems. IEEE Photonics Technol. Lett..

[3] Zvanovec S., Chvojka P., Haigh P.A., Ghassemlooy Z. (2015). Visible light communications towards 5G. Radioengineering.

[4] Le Minh H., O’Brien D., Faulkner G., Zeng L., Lee K., Jung D., Oh J., Won E.T. (2009). 100-Mb/s NRZ visible light communications using a postequalized white LED. IEEE Photonics Technol. Lett..

[5] Sung J.Y., Chow C.W., Yeh C.H. (2014). Is blue optical filter necessary in high speed phosphor-based white light LED visible light communications?. Opt. Express.

[6] Stepniak G., Schueppert M., Bunge C.A. (2015). Advanced modulation formats in phosphorous LED VLC links and the impact of blue filtering. J. Lightwave Technol..

[7] Piprek J. (2010). Efficiency droop in nitride-based light-emitting diodes. Phys. Status Solidi A.

[8] Schubert E.F. (2006). Light Emitting Diodes.

[9] Kamalakis T., Walewski J.W., Mileounis G. (2011). Empirical Volterra-series modeling of commercial light-emitting diodes. J. Lightwave Technol..

[10] Schüppert M., Bunge C.A. Characterization and equalization of nonlinearities in directly modulated resonant cavity light-emitting diodes. Proceedings of the 18th International Conference on Transparent Optical Networks (ICTON).

[11] Kamalakis T., Dede G. (2018). Nonlinear degradation of a visible-light communication link: A Volterra-series approach. Opt. Commun..

[12] Stepniak G., Siuzdak J., Zwierko P. (2013). Compensation of a VLC phosphorescent white LED nonlinearity by means of Volterra DFE. IEEE Photonics Technol. Lett..

[13] Hsu C.-W., Chen G.-H., Wei L.-Y., Chow C.-W., Lu I.-C., Liu Y.-L., Chen H.-Y., Yeh C.-H., Liu Y. (2017). Adaptive filtering for white-light LED visible light communication. Opt. Eng..

[14] Doyle F.J., Pearson K.R., Ogunnaike B.A. (2002). Determination of Volterra model properties. Identification and Control Using Volterra Models.

[15] Golub G.H., Loan C.F.V. (1996). Orthogonalization and Least squares. Matrix Computations.

[16] Proakis J.G., Manolakis D.G. (1996). Digital Signal Processing, Principles, Algorithms, Applications.

[17] Lee D.Q. (2012). Numerically Efficient Methods for Solving Least Squares Problems. http://www.math.uchicago.edu/~may/REU2012/REUPapers/Lee.pdf.

